# Communication is Key: Using Complex Co‐cultures to Decipher the Intercellular Crosstalk that Drives AD

**DOI:** 10.1002/alz70855_107155

**Published:** 2025-12-25

**Authors:** Katie Mao, Anders Smith, Veronica Roa, Maya Iskandar, Gerald Wambua, Doug Quackenbush, Joe Siefert, Lang Huynh, Brandon Taylor, Christopher Trussell, Andrew Gunderson, Frederick Lo, Youngjae Woo, Rebekah Decker, Michaela Kneissel, Jimmy Elliott

**Affiliations:** ^1^ Novartis Institute of Biomedical Research, San Diego, CA, USA; ^2^ Novartis Institute of Biomedical Research, Basel, Basel‐Stadt, Switzerland

## Abstract

**Background:**

Alzheimer's disease (AD) is complex, involving intercellular communications, molecular signaling, and direct interactions between multiple cell types. Studying these complicated interactions in humans is difficult as access to functional brain tissue is limited, for obvious ethical reasons. Nevertheless, a better understanding of the complex cellular interactions that contribute to AD will be critical for the discovery of novel therapeutics. Toward this goal, we aim to develop complex *in vitro* co‐culture models, capable of more accurately recapitulating the AD state, and illuminating the aberrant intercellular interactions involved in disease.

**Method:**

We are developing modular systems by independently deriving neurons, astrocytes, and microglia from induced pluripotent stem cells (iPSCs) using commercially available protocols, and combining them, as needed, into custom co‐culture systems. By independently perturbing individual cell types we will investigate how disease states alter cell‐cell communications using various methods such as RASL‐seq, snRNA‐seq, flow cytometry, phenotypic screens and functional assays. We hope to uncover specific elements of cellular communication that can be targeted to slow, halt, or even reverse disease progression.

**Result:**

We are thoroughly characterizing our iPSC‐derived neural cell types. In the case of our iPSC‐derived astrocytes and microglia, gene expression profiles were generated and compared to commercially available cell sources, and to data sets from human patients. Analysis confirmed varying degrees of similarity to reference patient samples, with our differentiated cells performing on par with or above the commercial ‘gold standard’. Using these well characterized monocultures, we have conducted preliminary co‐culture experiments. Astrocyte‐neuron co‐cultures showed improved neuronal maturation, neurite growth and network signaling compared to neuronal monoculture. In the case of astrocytes and microglia, perturbing only one cell type induced significant protein expression changes in in both cell types.

**Conclusion:**

Preliminary co‐culture experiments emphasized the interconnectedness of the various neural cell types and the great potential of complex co‐culture systems in understanding AD pathology. We believe that this work will contribute to the understanding of AD that will ultimately facilitate the development of effective therapeutic interventions.

